# Utility of Stellate Ganglion Block in Atypical Facial Pain: A Case Report and Consideration of Its Possible Mechanisms

**DOI:** 10.1155/2013/293826

**Published:** 2013-08-26

**Authors:** Harsha Shanthanna

**Affiliations:** Department of Anesthesiology, McMaster University, Health Sciences Centre 2U1, 1200 Main Street West, Hamilton, ON, Canada L8N 3Z5

## Abstract

We present this report of a young patient with chronic severe atypical facial pain who was successfully controlled with stellate ganglion block under ultrasound guidance. The patient had a history of severe disabling, unilateral, facial neuropathic pain with minimal response to analgesic medications. Upon assessment the patient had features suggestive of trigeminal neuralgia, although postherpetic neuralgia could not be ruled out. As a diagnostic test intervention, stellate ganglion block was tried under ultrasound guidance. The patient showed significant improvement in pain control and functional disability lasting beyond 10 weeks. Subsequent blocks reinforced the analgesia. Atypical facial pain has several differential diagnoses. The involvement of sympathetic system in its causation or sustenance is uncertain. Stellate ganglion block achieves sympathetic block of cervicofacial structures, and its blockade has been shown to affect chronic pain conditions. Although its mechanism is not clear, one has to consider its possible role in conditions of stress apart from directly controlling the sympathetic activity. There is certainly a role in exploring the potential benefits of stellate ganglion block in such clinical conditions. The technique of stellate block under ultrasound is also described, as it influences the safety and precision of the block.

## 1. Introduction

Stellate ganglion block (SGB) is commonly done for complex regional pain syndrome (CRPS). The underlying principle is to reset the altered sympathetic response. Considering the recent evidence, it is probably of lesser use in CRPS [1]. However there are several other indications for which SGB is considered to be beneficial. Atypical facial pain is considered when the facial pain has predominant features of trigeminal neuralgia but does not entirely fit the diagnostic criteria [2]. Psychological dimensions of chronic pain, such as stress and depression, form a common and important element of facial pain [3]. SGB has also shown benefits in conditions of stress, without any element of physiological pain. It is possible that there are significant clinical effects, not yet clearly understood, from SGB which are of benefit to patients with atypical facial pain. SGB must be considered as a test of therapeutic benefit to treatment resistant patients. Since the block involves injection near important vascular structures, the physician has to consider the safety and precision associated with the use of ultrasound.

## 2. Case Report

The patient was a young design engineer with a history of previous varicella zoster infection with severe pain affecting his right, lower half of his face in a nondermatomal fashion. The episodes of intense facial pain lasted minutes to hours with deep, burning, and lancinating type of quality. The pain typically involved the facial structures in the temporal area and lower part of face including front of the ear, jaw and chin. The pain was also associated with tearing of the eyes, blurred vision, and sensitivity to light. He was significantly disabled with marked restriction of daily activities, sleep, and depression. He was on disability after having quit his job. The excruciating pain had also made him significantly depressed. After having tried several groups of medications, including nonselective and selective antidepressants, carbamazepine, and gabapentin, he was presently being tried with long acting tramadol and lyrica with minimal success. The assessment of the patient revealed a young patient with features of flat effect. His face was unshaven without any obvious structural abnormalities. There was no identifiable swelling or change in color. Palpation revealed hyperalgesia and allodynia in the lower half of the face, centred in front of his ear and extending below the mandible. There was no associated headache. Rest of examination did not reveal any other findings. He had undergone an MRI which was normal and showed no abnormality with respect to trigeminal ganglion. Although it was not clear, it predominantly involved the lower 2 components of the trigeminal nerve (V2 and V3). As indicated by the above clinical findings we suspected the possibility of trigeminal neuralgia or a postherpetic neuralgia.

This patient was quiet involved in his treatment and had made significant efforts to collect and go through the medical literature pertaining to his clinical condition and the possible role of SGB and other interventions. Since the clinical picture did reveal features of trigeminal neuralgia, we considered the possibility of diagnostic trigeminal nerve block. However a diagnostic block of the stellate ganglion was initially done considering the relative ease compared to a fluoroscopy guided trigeminal nerve block. It was done under ultrasound guidance in an in-plane approach, using a high resolution probe (GE Ultrasound, LOGIQ e machine), with a 50 mm echostim needle (Benlan, Ontario, Canada). The solution used was 0.25% bupivacaine (5 mL) mixed with 8 mg of dexamethasone. Within minutes the patient showed signs of horner's syndrome and also noticed that his sensitivity over the right side had come down. He reported moderate to good pain relief lasting 10 weeks after the first injection. Similar injections were repeated and we noticed that there was an increase in the duration of pain-free period lasting nearly 3 months. His overall functioning improved. The intensity of pain decreased to 30% from 90%. Presently the patient continues to have pain relief lasting months and gets the SGB done intermittently when the condition flares up. He continues to be on the lyrica 150 mg, 2 times a day, and is using his tramadol on an occasional basis instead of the regular use.

## 3. Discussion

**Figure 1:**
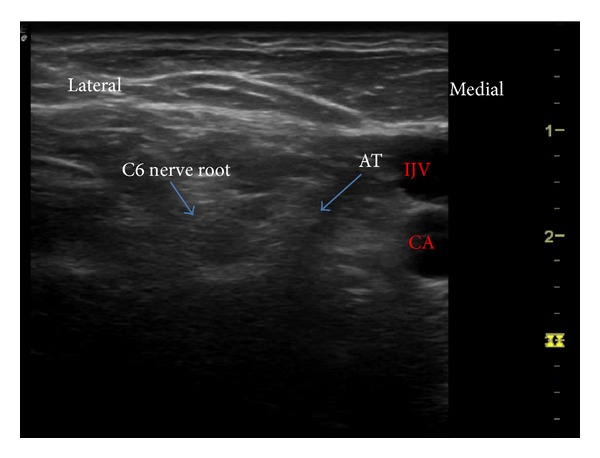
Identification of C6 level. IJV: internal jugular vein, CA: carotid artery.

**Figure 2:**
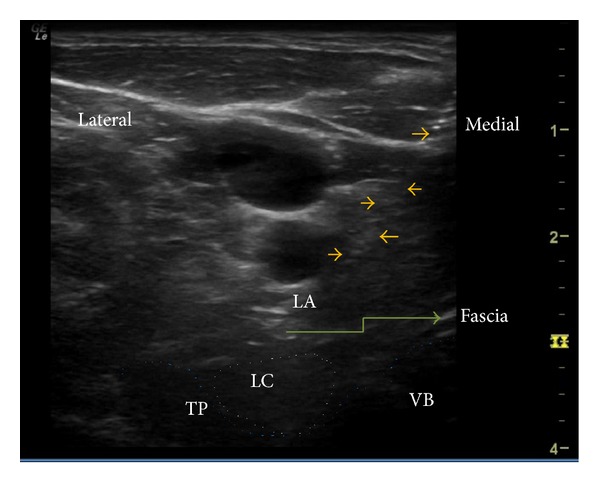
Stellate ganglion block in an in-plane approach with the needle lying on the prevertebral fascia. Needle is shown by the arrow marks, LC: longus colli, TP: transverse process, VB: vertebral body, LA: local anesthetic.

Our report demonstrates that SGB can successfully act in conditions of atypical facial pain with predominant features of trigeminal neuralgia involving the lower 2 segments (V2 and V3). Although the mechanism of action is not clear, this block should be considered in patients not responding to conservative management. Atypical facial pain is not actually a diagnosis; failure to appropriately diagnose some chronic facial pain conditions into previously known neuralgic conditions, with uniquely identifiable characteristics, allows the physicians to loosely use this term for reference [4]. International Association for Study of Pain (IASP) does not include this in the list of chronic pain conditions [5]. Facial pain can be excruciatingly severe and commonly causes significant distress and leads to depression in affected individuals [2]. Stellate ganglion, also called the cervicothoracic sympathetic ganglion, is present at the C7 vertebral level, lying at the neck of first rib, on top of the cervical prevertebral fascia. The sympathetic fibres for lower face and cervical and upper limb pass through this ganglion and are susceptible to be blocked with SGB. SGB has been widely used for CRPS, both as diagnostic of sympathetically mediated pain and also for possible therapy. However recent systematic review examining its role in CRPS is not fully supportive of its benefit [1]. Apart from CRPS, SGB has been successfully used in posttraumatic stress disorder, breast cancer pain, facial pain, refractory angina, vasculitis, herpes zoster, and other conditions. People have questioned the ability of sympathetic blockade achieved through SGB to provide pain relief. In a study by Schürmann et al., pain relief along with signs of sympathetic block was achieved in only 7 among 33 patients [6]. There is evidence that orofacial pain could involve sympathetic nervous system [7]. However there have been other mechanisms suggested for the clinical benefit observed with SGB. In rabbits, SGB decreased the nociceptive responses elicited by formalin injection possibly by reduction of substance P in the spinal cord and decreased plasma catecholamine release [8]. It is also postulated that SGB can be beneficial in a broad list of so many indications by its ability to normalize the melatonin release disorder [9, 10]. However, SGB has been reported to work quiet effectively in conditions of PTSD and anxiety disorders [11]. It is accepted that modulation of autonomic system can work in psychological conditions [3]. Although it is not plausible as an explanation of its mechanism in facial pain, we feel that there could be a major contribution from the psychological distress associated with the chronic facial pain, the modification of which could result in better pain control. Indeed a recent randomized trial has shown possibly better results with SGB in postherpetic neuralgia [12]. It is also to be observed that a trial of SGB in the early stages could provide better results with facial pain [13]. Our SGB was performed under ultrasound guidance (Figures 1 and 2). It has been shown that US guided SGB is probably safer and more precise [14]. Only 2–6 mls of solution could be necessary with appropriate needle positioning and spread [15]. Apart from making it safe by visualizing the vascular structures, it helps us locate the needle tip which needs to lie superficial to the longus colli muscle and not within it. The blind technique involves “the slight withdrawal after hitting the bone,” which is a subjective movement and hence may not be technically successful.
